# Supplementary material for: Coevolution of male and female mate choice can destabilize reproductive isolation
Source: Nat Commun. 2019 Nov 12;10:5122. doi: 10.1038/s41467-019-12860-9 (PMC6851176; doi:10.1038/s41467-019-12860-9)
Supplement: Supplementary file 4 — Description of Additional Supplementary Files [file 41467_2019_12860_MOESM4_ESM.pdf]

Description of Additional Supplementary Files

File Name: Supplementary Software 1

Description: Compressed file containing the source code for running the simulations and the analyses.
